# Roe, Interrupted: Estrogen Exposure Impairs Fish Fertility

**DOI:** 10.1289/ehp.112-a1010b

**Published:** 2004-12

**Authors:** Angela Spivey

Major research efforts have shown that endocrine disruptors—environmental chemicals that can interfere with the endocrine system—may affect reproduction of wildlife and even humans. Studies in fish, for example, have shown that endocrine disruptors can reduce sperm count, induce both male and female gonadal tissue or intermediate sexual characteristics in the same individual, and induce female-specific proteins in males. But little evidence to date has elucidated the effect of such changes on fertility. This month, Jon Nash of the Katholieke Universiteit Leuven in Belgium and colleagues report that long-term exposure to low concentrations of a synthetic estrogen may severely undermine the breeding success of wildlife populations, chiefly by producing sexually compromised males who disrupt breeding dynamics **[*EHP* 112:1725–1733]**.

Using zebrafish because of their short generation time, the researchers measured effects of exposure over three generations. They began with 720 fish divided into 60 groups of 12. The team recreated natural conditions in the aquaria to optimize fish breeding, and eggs were collected each day.

After a baseline assessment of egg numbers and egg viability (a cumulative statistic of unfertilized eggs and embryo mortality), the researchers exposed different groups to environmentally relevant concentrations of various estrogens: 5.0 nanograms per liter (ng/L) of the endogenous estrogen estradiol or either 0.5, 5.0, or 50.0 ng/L ethynylestradiol, a potent synthetic estrogen used in oral contraceptives. A control group received no exposure.

Except for the highest concentration of ethynylestradiol, none of the estrogen treatments affected egg numbers or egg viability in the baseline generation. Nor did any of the treatments affect survival of the eggs spawned by this generation.

But after 210 days (a full zebrafish lifetime) of exposure to the middle dose of 5.0 ng/L ethynylestradiol, the second generation of fish showed reduced fertility. None of the male fish in the second generation had normal testes, and they did not produce expressible sperm, although the females were fertile. None of this generation’s progeny survived beyond 14 hours postfertilization. In almost 12,000 eggs spawned, none were viable.

When two healthy, nonexposed males were added to the populations that had experienced reproductive failure, embryos began surviving. But the embryos’ rate of survival was still significantly less than in the control group. After close observation of the spawning in these tanks, the researchers found that the infertile males showed normal reproductive behavior, chasing the spawning females and competing with the fertile males for access. The researchers suggest that the reduced fertilization was caused at least in part by the compromised males interfering with the fertilization capability of the healthy males.

The researchers say their data show that development of the testes is more sensitive to disruption by ethynylestradiol than is reproductive behavior. Yet the relatively higher threshold of sensitivity of behavioral disruption may in fact produce stronger population-level consequences, as infertile males have a greater ability to interfere with breeding dynamics. They conclude that more information about the effects of endocrine disruptors on the interactions between fish in a spawning group is needed before the population-level effects of endocrine disruption can be understood.

